# Optimizing the Profile of [^99m^Tc]Tc–NT(7–13) Tracers in Pancreatic Cancer Models by Means of Protease Inhibitors

**DOI:** 10.3390/ijms21217926

**Published:** 2020-10-26

**Authors:** Panagiotis Kanellopoulos, Berthold A. Nock, Eric P. Krenning, Theodosia Maina

**Affiliations:** 1 Molecular Radiopharmacy, INRASTES, NCSR “Demokritos”, 15341 Athens, Greece; nock_berthold.a@hotmail.com; 2 Molecular Pharmacology, School of Medicine, University of Crete, Heraklion, 70013 Crete, Greece; 3 Cyclotron Rotterdam BV, Erasmus MC, 3015 CE Rotterdam, The Netherlands; erickrenning@gmail.com

**Keywords:** neurotensin subtype 1 receptor, neurotensin, theranostics, pancreatic cancer, [^99m^Tc]Tc radiotracer, neprilysin, Entresto, angiotensin_converting enzyme, lisinopril, clinical translation

## Abstract

Background: The overexpression of neurotensin subtype 1 receptors (NTS1Rs) in human tumors may be elegantly exploited for directing neurotensin (NT)-based radionuclide carriers specifically to cancer sites for theranostic purposes. We have recently shown that [^99m^Tc]Tc–DT1 ([^99m^Tc]Tc–[N_4_–Gly^7^]NT(7–13)) and [^99m^Tc]Tc–DT5 ([^99m^Tc]Tc–[N_4_–*β*Ala^7^,Dab^9^]NT(7–13)) show notably improved uptake in human colon adenocarcinoma WiDr xenografts in mice treated with neprilysin (NEP) inhibitors and/or angiotensin-converting enzyme (ACE) inhibitors compared with untreated controls. Aiming toward translation of this promising approach in NTS1R-positive pancreatic ductal adenocarcinoma (PDAC) patients, we now report on the impact of registered NEP/ACE inhibitors on the performance of [^99m^Tc]Tc–DT1 and [^99m^Tc]Tc–DT5 in pancreatic cancer models. Methods: The cellular uptake of [^99m^Tc]Tc–DT1 and [^99m^Tc]Tc–DT5 was tested in a panel of pancreatic cell lines, and their stability was assessed in mice treated or not treated with Entresto, lisinopril, or their combinations. Biodistribution was conducted in severe combined immunodeficiency (SCID) mice bearing pancreatic AsPC-1 xenografts. Results: The Entresto + lisinopril combination maximized the metabolic stability of the fast-internalizing [^99m^Tc]Tc–DT1 in mice, resulting in notably enhanced tumor uptake (7.05 ± 0.80% injected activity (IA)/g vs. 1.25 ± 0.80% IA/g in non-treated controls at 4 h post-injection; *p* < 0.0001). Conclusions: This study has shown the feasibility of optimizing the uptake of [^99m^Tc]Tc–DT1 in pancreatic cancer models with the aid of clinically established NEP/ACE inhibitors, in favor of clinical translation prospects.

## 1. Introduction

The neurotensin subtype 1 receptor (NTS1R) has been regarded as a valid biomolecular target in cancer theranostics, owing to its high-density expression in a variety of human tumors, such as pancreatic ductal adenocarcinoma (PDAC), colon carcinoma, Ewing’s sarcoma, or prostate and breast cancer [1,2,3,4,5,6,7,8,9,10,11,12]. This overexpression can be elegantly exploited to direct NTS1R-seeking radionuclide carriers to tumor sites [13,14]. Typically, radiolabeled analogs of the C-terminal hexapeptide fragment of neurotensin (NT = Pyr–Leu–Tyr–Glu–Asn–Lys–Pro–Arg–Arg–Pro–Tyr–Ile–Leu–OH), NT(8–13), have been considered for such purposes [15,16,17,18,19,20,21,22,23,24]. For stable binding of metal radionuclides, a suitable chelator needs to be covalently attached to the N-terminus of the peptide chain, directly or via a linker [25].

Accordingly, NT conjugates can be properly designed to carry a gamma emitting radiometal ([^99m^Tc]Tc, [^111^In]In) for single-photon emission computed tomography (SPECT), or a positron emitter ([^68^Ga]Ga, [^64^Cu]Cu) for positron emission tomography (PET). SPECT or PET imaging of tumor sites allows for the diagnosis, staging, and assessment of disease spread, and indicates patients eligible for radionuclide therapy. During radionuclide therapy, the respective therapeutic radiometal (beta emitter: [^177^Lu]Lu, [^90^Y]Y; Auger electron emitter: [^111^In]In; alpha emitter: [^225^Ac]Ac) will be delivered to NTS1R-positive cancer sites applying the same carrier molecule. The success of the aforementioned theranostic concept has already been established in the clinic for radiolabeled somatostatin analogs used in the management of patients with somatostatin receptor-positive neuroendocrine tumors (NETs) [14,26,27]. Radiolabeled, prostate-specific membrane antigen (PSMA) inhibitors recently introduced against prostate cancer represent another successful paradigm of theranostics in nuclear oncology [14,27].

We have previously developed a small library of NT(7–13) analogs, coupled to an acyclic tetraamine chelator at the N-terminus for stable binding of the eminent SPECT radionuclide [^99m^Tc]Tc. The preclinical evaluation of this series of compounds was carried out in human colon adenocarcinoma HT29 and WiDr cells expressing the NTS1R [28,29]. During translation of the best-performing radiotracer, [^99m^Tc]Tc–DT6 ([^99m^Tc]Tc–[N_4_–*β*Ala^7^,Dab^9^,Tle]NT(7–13); N_4_ = 6–(carboxy)–1,4,8,11–tetraazaundecane), in a small number of patients, poor NTS1R targeting of tumor lesions and very fast washout of radioactivity via the kidneys into urine was observed [30]. This disappointing result was attributed to rapid in vivo catabolism of the linear peptide chain. In fact, several proteases have been reported to swiftly degrade NT and its analogs. Thus, angiotensin-converting enzyme (ACE) has been shown to rapidly cleave the Tyr^11^–Ile^12^ bond, whereas neprilysin (NEP) quickly hydrolyzes both the Pro^10^–Tyr^11^ and the Tyr^11^–Ile^12^ bonds [31,32,33,34,35,36]. Two additional metallopeptidases, EC 3.4.24.15 (thiolsensitive metallo-oligopeptidase, thimet-oligopeptidase (TOP)) cleaving the Arg^8^–Arg^9^ bond and EC 3.4.24.16 (neurolysin) hydrolyzing the Pro^10^–Tyr^11^ bond, may further contribute in the degradation of NT and its analogs [37,38]. However, these proteases are located within cells, and hence are less expected to encounter circulating NT radioligands and disturb their delivery to tumor sites.

In a recent study, we were able to demonstrate the involvement of ACE and NEP in the in vivo degradation of [^99m^Tc]Tc–DT1 ([^99m^Tc]Tc–[N_4_–Gly^7^]NT(7–13)) and [^99m^Tc]Tc–DT5 ([^99m^Tc]Tc–[N_4_–*β*Ala^7^,Dab^9^]NT(7–13)) [39]. Furthermore, we showed that co-injection of the NEP inhibitor phosphoramidon (PA) [40] and the ACE inhibitor lisinopril (Lis) [41] resulted in significant stabilization of these radiotracers in peripheral mouse blood. Consequently, the uptake of both [^99m^Tc]Tc–DT1 and [^99m^Tc]Tc–DT5 in human NTS1R-positive colon adenocarcinoma tumors in mice was significantly enhanced [39]. Following this rationale, we are now interested in translating these positive findings into pancreatic cancer models. It should be noted that 95% of all pancreatic cancers are actually PDAC, one of the most devastating cancer types, with a five-year survival not exceeding 10%. A major problem in the management of PDAC is that the disease remains practically asymptomatic, and has already disseminated by the time of diagnosis. Treatment may be comprised of surgical resection followed by chemo/radiotherapy, but outcomes are rather limited, with most patients dying soon after diagnosis and the average survival not surpassing 28 months [42]. It is therefore imperative to make available effective theranostic tools to combat PDAC, including radiolabeled NTS1R-directed probes.

In the present study, we first evaluated the uptake of [^99m^Tc]Tc–DT1 in a series of commercially available pancreatic cell lines, namely AsPC-1, PANC-1, MiaCapa-2, and Capan-1. We were interested in identifying the cell line by combining high radioligand uptake, indicative of high NTS1R expression, with practical convenience of handling, including acceptable doubling times during culture, as well as easy development of experimental tumors in severe combined immunodeficiency (SCID) mice available in our facilities. The cell uptake of [^99m^Tc]Tc–DT1 and [^99m^Tc]Tc–DT5 was compared in the AsPC-1 cell line of choice. The metabolic stability of the two radioligands was compared in healthy mice without or during treatment with two approved and registered drugs, Entresto [43,44] or Lis, or their combination. Entresto pills for oral use contain a pro-form of the potent NEP inhibitor sacubitril released in vivo (Figure S1, Supplementary Materials), while Lis is a potent ACE inhibitor [41]. The impact of these treatments on the radioligand tumor uptake was studied in SCID mice bearing AsPC-1 xenografts, in order to assess the translational prospects of this methodology in PDAC patients.

## 2. Results

### 2.1. Radiolabelling and Quality Control

**Figure 1:**
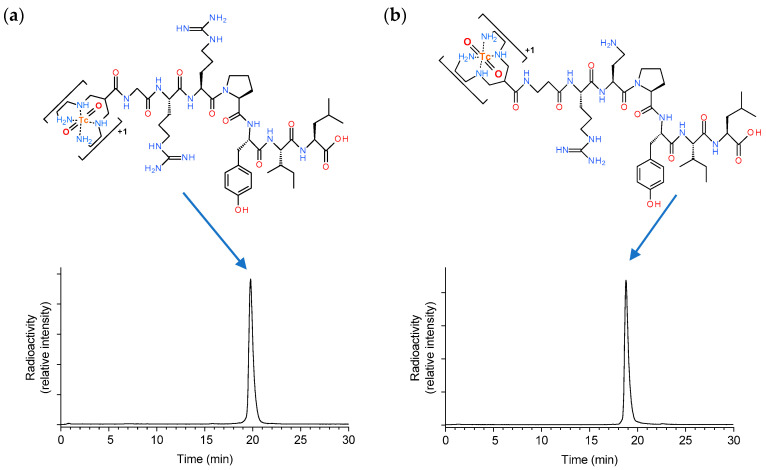
Representative radiochromatograms of high-performance liquid chromatography (HPLC) analysis of the radiolabeling mixture of (**a**) [^99m^Tc]Tc–DT1 and (**b**) [^99m^Tc]Tc–DT5 with the respective radioligand structures indicated.

Radiolabelling of DT1 and DT5 with [^99m^Tc]Tc was accomplished by 30 min incubation at room temperature in alkaline aqueous medium containing citrate anions and SnCl_2_ as a reductant. Quality control of the radiolabelled products included high-performance liquid chromatography (HPLC) and instant thin-layer chromatography (ITLC) analysis, and revealed less than 2% total radiochemical impurities ([^99m^Tc]TcO_4_^−^, [^99m^Tc]Tc citrate, and [^99m^Tc]TcO_2_ × nH_2_O). A single radiopeptide species was obtained at molecular activities of 20–40 MBq [^99m^Tc]Tc/nmol peptide; representative radiochromatograms are included in Figure 1. Based on these findings, [^99m^Tc]Tc–DT1 and [^99m^Tc]Tc–DT5 were used without further purification in all subsequent biological assays.

### 2.2. Cell Uptake Studies

#### Comparative Cell Uptake of [^99m^Tc]Tc–DT1 in AsPC-1, PANC-1, MiaCapa-2, and Capan-1 Cells

**Figure 2:**
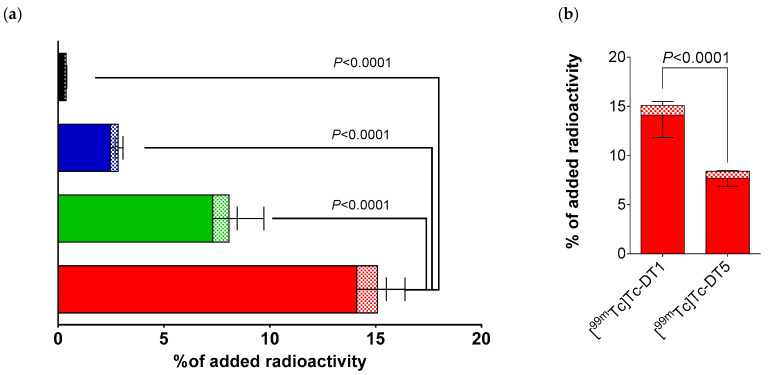
(**a**) Neurotensin subtype 1 receptor (NTS1R)-specific cell uptake of [^99m^Tc]Tc–DT1 in AsPC-1 (red column), PANC-1 (green column), MiaCapa-2 (blue column), and Capan-1 (black column) cells during 1 h incubation at 37 °C. (**b**) Comparison of NTS1R-specific uptake of [^99m^Tc]Tc–DT1 and [^99m^Tc]Tc–DT5 in AsPC-1 cells during 1 h incubation at 37 °C; solid bars: internalized fraction; checkered bars: membrane-bound fraction. Results represent average values ± SD (*n* = 3, in triplicate); non-specific values were obtained in the presence of 1 μM neurotensin (NT), and were subtracted from totals to provide specific values. The study was conducted with cells as confluent monolayers.

During 1 h incubation at 37 °C, [^99m^Tc]Tc–DT1 displayed distinct NTS1R-mediated uptake across the AsPC-1, PANC-1, MiaCapa-2, and Capan-1 cell lines, as summarized in Figure 2a. The rank of specific cell uptake of [^99m^Tc]Tc–DT1 was AsPC-1 (15.2% ± 2.6%) > PANC-1 (8.1% ± 2.8%; *p* < 0.0001 vs. AsPC-1) > MiaCapa-2 (2.7% ± 0.3%; *p* <0.0001 vs. PANC-1) > Capan-1 (0.4% ± 0.1%; *p* < 0.0001 vs. PANC-1, *p* > 0.05 vs. MiaCapa-2). In all cases, the bulk of cell-bound radioactivity was found in the internalized fraction, as consistent with a radioagonist profile. Cell uptake was banned in the presence of excess NT, implying an NTS1R-mediated process (Table S1; Supplementary Materials).

When comparing [^99m^Tc]Tc–DT1 and [^99m^Tc]Tc–DT5 for their NTS1R-uptake in AsPC-1 cells at 1 h incubation at 37 °C, [^99m^Tc]Tc–DT1 displayed significantly higher values (15.2% ± 2.6% vs. 8.4% ± 0.8%; *p* < 0.0001), as depicted in Figure 2b.

**Figure 3:**
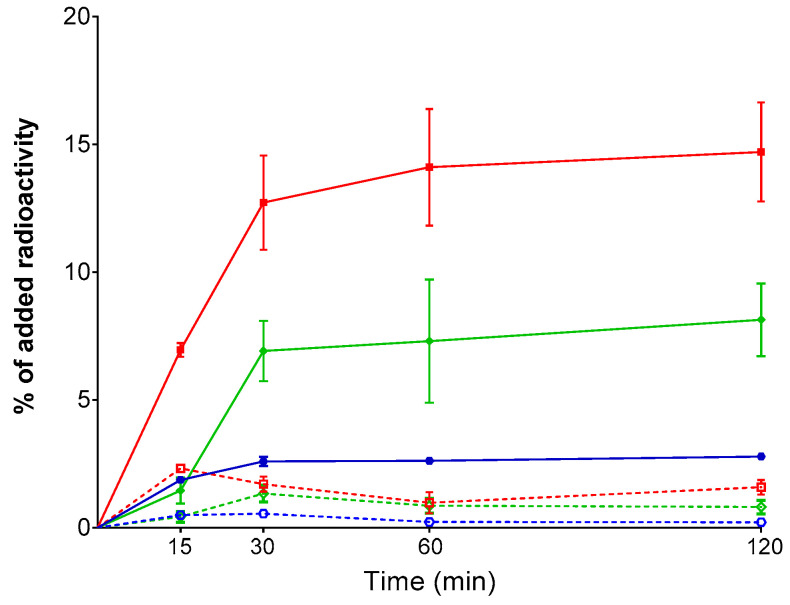
Time-dependent, NTS1R-specific cell uptake curves of [^99m^Tc]Tc–DT1 in AsPC -1 (red lines), PANC-1 (green lines), and MiaCapa-2 (blue lines) cells at 37 °C (solid lines: internalized fraction; dotted lines: membrane-bound fraction). Results represent average values ± SD (*n* = 3, in triplicate); non-specific values were obtained in the presence of 1 μM NT, and were subtracted from totals to provide specific values. The study was conducted with cells as confluent monolayers.

Time-dependent cell uptake curves for [^99m^Tc]Tc–DT1 in AsPC-1, PANC-1, and MiaCapa-2 cells are included in Figure 3, but not for Capan-1, cells due to their poor overall uptake during the 1 h incubation. We observed the same trend of [^99m^Tc]Tc–DT1 uptake across cell lines at all time intervals. Likewise, the bulk of radioactivity was found in the internalized fraction, with a lesser amount bound on the cell membrane.

### 2.3. In Vivo Studies

#### 2.3.1. Comparative Stability of [^99m^Tc]Tc–DT1 and [^99m^Tc]Tc–DT5 in Mice: The Impact of Protease Inhibitors

**Figure 4:**
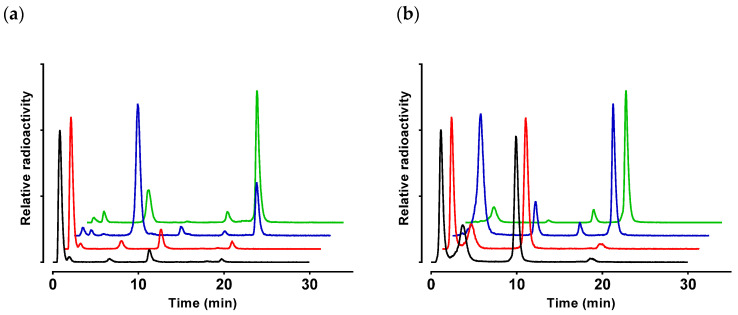
Representative radiochromatograms of HPLC analysis of mouse blood samples collected 5 min pi with (**a**) [^99m^Tc]Tc–DT1 and (**b**) [^99m^Tc]Tc–DT5; black line graphs correspond to samples from untreated controls, red line graphs to samples from animals receiving per os Entresto 30 min prior to radioligand injection, blue line graphs to samples from animals with Lis co-injected together with the radioligand, and green line graphs to samples from animals receiving Entresto 30 min prior to radioligand co-injection with Lis (HPLC system 2); percentages of intact radioligand are summarized in Table 1.

The radiotracers exhibited poor resistance to degrading proteases after intravenous injection in mice. As revealed by HPLC analysis of blood samples collected at 5 min post-injection (pi), [^99m^Tc]Tc–DT1 and [^99m^Tc]Tc–DT5 degraded equally fast (1.8 ± 0.8% and 1.2 ± 0.2% intact; *p* > 0.05), although the pattern of forming radiometabolites was different for each compound (Table 1; Figure 4).

Treatment of mice with clinically established NEP and ACE inhibitors, as well as their combination, exerted profound effects not only on the overall metabolic stability of [^99m^Tc]Tc–DT1 and [^99m^Tc]Tc–DT5, but also on the pattern of radiometabolites found in mice blood at 5 min pi. Thus, treatment of mice with Entresto taken orally 30 min prior to radiotracer injection had hardly any effect on stability (*p* > 0.05 vs. controls for both radioligands). Conversely, co-injection of Lis significantly increased the overall stability of both radiotracers ([^99m^Tc]Tc–DT1 to 18.8 ± 2.5% intact, *p* < 0.0001 vs. controls; [^99m^Tc]Tc–DT5 to 28.7 ± 3.6% intact, *p* < 0.0001 vs. controls). Interestingly, a combination of these treatments resulted in further significant increases of metabolic stabilities of the two radiotracers ([^99m^Tc]Tc–DT1 to 63.8 ± 7.5% intact, *p* < 0.0001 vs. Lis-treated mice; [^99m^Tc]Tc–DT5 to 70.2 ± 4.9% intact, *p* < 0.0001 vs. Lis-treated mice).

These results imply cooperation of the two proteases, ACE and NEP, in the fast in vivo catabolism of [^99m^Tc]Tc–DT1 and [^99m^Tc]Tc–DT5. Furthermore, they indicate that ACE acts faster, given that Entresto alone provides no considerable improvement of stability of either radiotracer, and is only effective during concomitant in situ inhibition of ACE by Lis.

#### 2.3.2. Biodistribution of [^99m^Tc]Tc–DT1 in AsPC-1 Tumor-Bearing SCID Mice: The Impact of Protease Inhibitors

The effect of in situ NEP + ACE inhibition on the biodistribution of [^99m^Tc]Tc–DT1 in SCID mice bearing AsPC-1 xenografts at 4 h pi is summarized in Table 2; data is expressed as percent of injected activity per gram tissue (% IA/g) and represents mean values ± SD. The [^99m^Tc]Tc–DT5 was not included in this study, in view of its inferior uptake by AsPC-1 cells compared with [^99m^Tc]Tc–DT1 (Figure 2b). In addition, it displayed high renal values in mice during a previous study, most probably as a result of the pendant *δ*-amine of Dab^9^ in the peptide chain [39].

We observe that the radioactivity in the blood and the body of mice has substantially cleared at 4 h pi. Uptake in the AsPC-1 xenografts is clearly visible, albeit low (1.25 ± 0.14% IA/g), while a higher radioactivity level is retained in the kidneys (4.18 ± 3.80% IA/g). Treatment of mice with the Entresto + Lis combination led to notable increase of the [^99m^Tc]Tc–DT1 uptake in the AsPC-1 tumors (to 7.05 ± 0.80% IA/g; *p* < 0.0001 vs. controls) without affecting background radioactivity levels, except for the kidneys. Although some minor increase is observed in the kidneys, the increase in the tumors is higher during treatment, resulting in Tu/Ki ratios > 1. The observed high uptake of [^99m^Tc]Tc–DT1 in the AsPC-1 tumors during Entresto + Lis treatment was shown to be NTS1R-mediated, given that it was banned by co-injection of excess NT in mice treated with the same inhibitor regimen (drop to 0.74 ± 0.01% IA/g; *p* < 0.0001 vs. Entresto + Lis treated mice).

The uptake of [^99m^Tc]Tc–DT1 at 4 h pi in MiaCapa-1 xenografts in SCID mice treated with the Entresto + Lis combination (Table S2; Supplementary Materials) was much lower compared with AsPC-1 tumors in the respective Entresto + Lis group (1.97% ± 0.23% IA/g vs. 7.05% ± 0.80% IA/g; *p* < 0.0001), in line with findings from in vitro cell uptake/internalization assays.

## 3. Discussion

The limited success in the application of NTS1R-directed NT(8-13)-derived radiopeptides in cancer theranostics [30,45,46] has been related to the rapid cleavage of the NT backbone by peptidases. For example, ACE has been reported to hydrolyze the Tyr^11^–Ile^12^ bond [31,32], and its action was very soon recognized during metabolic stability determination assays [24]. Typically, this kind of experiments involves in vitro incubation of radioligands in mouse/human plasma, followed by chromatographic analysis of incubate samples to detect generated radiometabolites. Consequently, efforts toward ACE-resistant NTS1R-radioligands were directed to stabilize the Tyr^11^–Ile^12^ bond, primarily via Tle^12^/Ile^12^–replacement [22,24,29].

Following this rationale, we have developed the Tle^12^/Ile^12^–modified [^99m^Tc]Tc–DT6, displaying high receptor affinity (IC_50_ = 0.08 ± 0.02 nM) and high stability in mice plasma (>90% intact in mice plasma 2 h incubates) [29]. Unexpectedly, however, [^99m^Tc]Tc–DT6 failed to target NTS1R-expressing tumor lesions in a subsequent “proof-of-principle” study in patients [30]. Aiming to elucidate these findings, we further studied the cell binding capabilities and in vivo stability of the radiotracer. We observed that the Tle^12^/Ile^12^ substitution led to poor binding/internalization of [^99m^Tc]Tc–DT6 in WiDr cells compared with non-Ile^12^-substituted radioligands (e.g., 1.0 ± 0.4% specific cell binding vs. 10.1 ± 2.3% of [^99m^Tc]Tc–DT1 at 1 h incubation; *p* < 0.0001) [39]. Furthermore, the in vivo stability of [^99m^Tc]Tc–DT6 was found to be surprisingly lower than expected from in vitro assays (only 55.1 ± 3.9% of the radiotracer detected intact in peripheral mice blood at 5 min pi). The combination of these two unfavorable features seems to have led to the sub-optimal performance of [^99m^Tc]Tc–DT6 in patients.

Earlier reports have implicated NEP (next to ACE) in the in vivo catabolism of NT, rapidly hydrolyzing both the Tle^12^–Ile^12^ and the Pro^10^–Tyr^11^ bonds of the peptide chain [31,32,35]. NEP is an ecto-enzyme with a broad substrate repertoire, and is omnipresent on vasculature walls and major tissues/organs of the body in high local concentrations, where it remains anchored on the membrane of epithelial cells [47,48]. Consequently, during stability assessment of peptide radioligands by in vitro assays in plasma incubates, the fast degrading action of NEP documented in vivo has been altogether missed. We have shown that NEP can quickly break down a wide range of radiopeptides entering the circulation, thereby compromising their supply and accumulation on tumor models in mice. Next, we were able to drastically interfere with this chain of disadvantageous events with the aid of NEP inhibitors. Through in situ inhibition of NEP, we could induce marked enhancement of tumor uptake of numerous biodegradable radiopeptides [49,50].

In the present study, we were interested in exploring the efficacy of [^99m^Tc]Tc–DT1 to target NTS1R–positive pancreatic cancer during NEP and ACE inhibition in mice models. We have selected [^99m^Tc]Tc–DT1 as the radioligand because it displayed maximum internalization/cell-binding amongst a series of related NT(7–13)-based radiotracers in colon adenocarcinoma WiDr cells [39]. As a first task, we have investigated the cell-binding/internalization of [^99m^Tc]Tc–DT1 in a panel of human pancreatic cancer cell lines, in order to determine the cell line of choice to use in our preclinical models. As shown in Figure 2, clear differences could be established in the cell uptake/internalization of [^99m^Tc]Tc-DT1 across AsPC-1, PANC-1, MiaCapa-2, and Capan-1 cells, with highest values associated with AsPC-1 cells at 1 h incubation. It is interesting to note that our findings were in line with previously reported NTS1R expression at the gene and protein levels across these cell lines [51]. Next, a time-dependent internalization study was carried out, whereby [^99m^Tc]Tc–DT1 displayed again significantly higher values in AsPC-1 cells compared with all other cell lines (Figure 3). Subsequently, we compared the cell uptake/internalization of [^99m^Tc]Tc–DT1 and a second, non-Tle^12^/Ile^12^-modified reference, [^99m^Tc]Tc–DT5. In agreement with findings from WiDr cells [39], [^99m^Tc]Tc–DT1 showed significantly higher values than [^99m^Tc]Tc–DT5 in AspC-1 cells (Figure 2b).

Our second goal was to stabilize [^99m^Tc]Tc–DT1, and the second reference [^99m^Tc]Tc–DT5, in mice circulation by in situ ACE and/or NEP inhibition, using clinically established inhibitors. Through co-injection with the NEP inhibitor PA or the ACE-inhibitor Lis, we could previously induce significant stabilization of these radioligands in peripheral mice blood [39]. PA is not a clinically approved drug [40], and consequently time- and cost-intensive studies are required for approval of its use in a “proof-of-concept” study in PDAC patients. For such purposes, we have instead selected Entresto, a registered anti-hypertensive drug containing sacubitril (AHU377) [43,44]. The latter is actually the pro-drug, releasing the potent and specific NEP inhibitor sacubitrilat (LBQ657) in vivo upon ester-hydrolysis by native esterases (Figure S1). We have orally administered single 12 mg/200 mL doses of Entresto (24/26 sacubitril/valsartan) pills per animal (*vide infra*) 30 min prior to radioligand injection to accomplish maximum NEP inhibition. As summarized in Table 1, both [^99m^Tc]Tc–DT1 and [^99m^Tc]Tc–DT5 were found to be >63% intact in peripheral mice blood during concomitant NEP- and ACE-inhibition, vs. the <2% intact found in non-treated controls. Entresto alone had only a very minor effect on stability, in contrast to Lis, which led to partial stabilization of the two analogs. However, Entresto had significant impact on further stabilizing the radiotracers when mice received Lis as well. This finding is consistent with the assumption that ACE acts faster on the two analogs, and upon ACE inhibition, NEP is given time to act. These stabilization effects validate Entresto as a viable candidate for clinical testing of this concept.

The third and final goal of the present study was to evaluate the biodistribution of [^99m^Tc]Tc–DT1 in pancreatic mice models without or during treatment with the Entresto + Lis combination. Based on cell uptake results, we selected AsPC-1 cells for tumor induction in mice. As summarized in Table 2, Entresto + Lis treatment resulted in marked increase of [^99m^Tc]Tc–DT1 uptake in the AsPC-1 xenografts at 4 h pi (7.05% ± 0.80% IA/g vs. 1.25% ± 0.14% IA/g in controls; *p* < 0.0001), which was shown to be NTS1R-mediated. Uptake in all other organs remained low, except for the kidneys. However, in the Entresto + Lis treated mice, the tumor-to-kidneys ratio was clearly superior. To our knowledge, these are the highest uptake values reported for AsPC-1 xenografts in mice for radiolabeled NT-radiotracers. For example, [^68^Ga]Ga–DOTA–NT–20.3 (Ac–Lys(1,4,7,10-tetraazacyclododecane–1,4,7,10-tetraacetic acid)–Pro–Me–Arg–Arg–Pro–Tyr–Tle–Leu–OH) reached 5.28% ± 0.93% IA/g uptake at 1 h pi, with about the same values found in the kidneys [52]. In another example, the bi-modal tracer [^68^Ga]Ga–NODAGA–Lys(Cy5**)–AEEAc–[Me–Arg^8^,Tle^12^]NT(7−13) (NODAGA: 1,4,7-triazacyclononane,1-glutaric acid-4,7-acetic acid; Cy5**: tetrasulfonated cyanine 5.0; AEEAc: 8-amino-3,6-dioxaoctanoic acid) reached 2.56% ± 0.97% IA/g at 1 h in the same model, with quite elevated kidney values though (20% IA/g) [53]. However, such comparisons should be made with caution, in view of potential differences in protocols applied across labs.

The aforementioned positive results obtained with the Entresto + Lis combination in AsPC-1 xenografts confirm previous observations in mice bearing WiDr tumors and treated with PA + Lis [39]. Undoubtedly, perspectives for clinical translation of this promising methodology in PDAC patients rely on the availability of clinically established NEP and ACE inhibitors, such as Entresto [43,44] and Lis [41] used in the present study. It should be noted that application of the NEP inhibition approach has been recently shown to improve the diagnostic sensitivity of radiolabeled gastrin in medullary thyroid cancer patients. In this first “proof-of-concept” study, patients received per os the registered and widely used anti-diarrhea drug racecadotril [54]. The latter is a pro-drug releasing the potent NEP-inhibitor thiorphan in the blood after in vivo ester-hydrolysis by native esterases [48]. Therefore, further studies are warranted to explore the applicability of this concept in PDAC patients as well.

## 4. Materials and Methods

### 4.1. Chemicals and Radionuclides

All chemicals were reagent grade, and were used as such without further purification. The peptide conjugates DT1 and DT5 were synthesized on the solid support and obtained from PiChem (Graz, Austria). NT was purchased from Bachem (Bubendorf, Switzerland). Entresto was obtained from a local pharmacy, and Lis was purchased from Sigma-Aldrich (St. Louis, MO, USA).

Technetium-99m in the form of [^99m^Tc]NaTcO_4_ was collected by elution of a [^99^Mo]Mo/[^99m^Tc]Tc generator (Ultra-Technekow V4 Generator, Curium Pharma, Petten, The Netherlands).

#### 4.1.1. Radiolabeling

The lyophilized peptide analogs were dissolved in water to a final concentration of 1 mM, and 50 μL aliquots were stored at −20 °C. Labelling with [^99m^Tc]Tc was performed in an Eppendorf vial containing 0.5 M phosphate buffer (pH 11.5; 50 µL). The [^99m^Tc]NaTcO_4_ eluate (420 µL, 370–550 MBq) was added to the vial, followed by 0.1 M sodium citrate (5 µL), the peptide stock solution (15 µL, 15 nmol), and a freshly prepared SnCl_2_ solution in ethanol (10 µL, 10 μg). The mixture was left to react for 30 min at room temperature, and the pH was neutralized with the addition of 0.1 M HCl.

#### 4.1.2. Quality Control

Quality control was comprised of radioanalytical, high-performance liquid chromatography (HPLC) and instant thin-layer chromatography (ITLC). HPLC analyses were performed on a Waters Chromatograph coupled to a 996-photodiode array UV detector (Waters, Vienna, Austria) and a Gabi gamma detector (Raytest RSM Analytische Instrumente GmbH, Straubenhardt, Germany). Data processing and chromatography were controlled with Empower Software (Waters, Milford, MA, USA). For analyses, a Symmetry Shield RP-18 (5 μm, 3.9 mm × 150 mm) cartridge column (Waters, Eschborn, Germany) was eluted at 1 mL/min flow rate with a linear gradient system, system 1, starting from 0% B and advancing to 40% B within 20 min (solvent A = 0.1% aqueous trifluoroacetic acid (TFA) and B = acetonitrile (MeCN)). ITLC analyses were performed on Whatman 3 mm chromatography paper strips (GE Healthcare, Chicago, IL, United States), developed up to 10 cm from the origin, with 5 M ammonium acetate/MeOH 1:1 (*v*/*v*) for the detection of reduced, hydrolyzed technetium ([^99m^Tc]TcO_2_ × nH_2_O) or acetone for the detection of [^99m^Tc]TcO_4_^−^.

All manipulations with beta- and gamma-emitting radionuclides and their solutions were performed by trained and authorized personnel behind suitable shielding in licensed laboratories, in compliance with European radiation safety guidelines, and were supervised by the Greek Atomic Energy Commission (license #A/435/17092/2019).

### 4.2. In Vitro Assays

#### 4.2.1. Cell Lines and Culture

The human pancreatic adenocarcinoma AsPC-1, PANC-1, MiaCapa-2, and Capan-1 cell lines were obtained from LGC Standards GmbH (Wesel, Germany). All culture reagents were purchased from Gibco BRL, Life Technologies (Grand Island, NY, USA) or from Biochrom KG Seromed (Berlin, Germany). Cells were grown in Roswell Park Memorial Institute-1640 Medium (RPMI-1640; AsPC-1 cells) or Dulbecco’s Modified Eagle’s Medium (DMEM; PANC-1, MiaCapa-2, and Capan-1 cells) with GlutaMAX-I, supplemented with 10% (*v*/*v*) fetal bovine serum (FBS), 100 U/mL penicillin, and 100 μg/mL streptomycin, and kept in a controlled humidified air containing 5% CO_2_ at 37 °C. Splitting of cells with a ratio of 1:3 to 1:5 was performed when approaching confluency, using a trypsin/EDTA (0.05%/0.02% *w*/*v*) solution.

#### 4.2.2. Internalization of [^99m^Tc]Tc Radiotracers in AsPC-1, PANC-1, MiaCapa-2, and Capan-1 Cells

For internalization assays, AsPC-1, PANC-1, MiaCapa-2, and Capan-1 cells were seeded in six-well plates (≈1 × 10^6^ cells per well) the day before the experiment. Cells were rinsed twice with ice-cold internalization medium (IM; culture medium supplemented by 1% (*v*/*v*) FBS), and then fresh IM was added (1.2 mL) at 37 °C, followed by test radiopeptide (250 fmol total peptide in 150 μL IPBS (0.5% *w*/*v* BSA–PBS,) 100,000–200,000 cpm). Non-specific internalization was determined by a parallel triplicate series containing 1 μM NT. After incubation at 37 °C, the plates were placed on ice, the medium was collected, and the plates were washed with IPBS (1 mL). Membrane-bound fractions were collected by incubating the cells 2 × 5 min in acid-wash solution (2 × 600 µL; 50 mM glycine buffer pH 2.8, 0.1 M NaCl) at room temperature. After rinsing the cells with IPBS (1 mL), internalized fractions were collected by lysing the cells with 1 M NaOH (2 × 600 µL). Sample radioactivity was measured on the gamma counter, and the percentage of specific internalized and membrane-bound fractions were calculated with Microsoft Excel (after subtracting the non-specific from the overall internalized and membrane-bound counts). Results represent the specific mean internalized ± SD of total added radioactivity per well from three experiments performed in triplicate. Furthermore, the internalization and cell uptake of [^99m^Tc]Tc-DT1 and [^99m^Tc]Tc-DT5 were compared head-to-head during 1 h incubation in AsPC-1 cells at 37 °C, following the above-described procedure.

In another set of experiments, the internalization/cell uptake of [^99m^Tc]Tc–DT1 was assessed overtime in AsPC-1, PANC-1, and MiaCapa-2 by incubation at 37 °C. During this study, the same protocol was followed again, but four different incubation times were applied: 15 min, 30 min, 1 h, and 2 h; Capan-1 cells were not included in this assay, due to the poor internalization/cell uptake of [^99m^Tc]Tc–DT1 shown in the first set of experiments at 1 h.

### 4.3. Animal Studies

#### 4.3.1. Metabolic Studies in Mice

A bolus containing [^99m^Tc]Tc–DT1 and [^99m^Tc]Tc–DT5 (100 μL, 50–60 MBq, 3 nmol of total peptide in vehicle: saline/EtOH = 9:1 *v*/*v*) was injected in the tail vein of healthy male Swiss albino mice, together with the vehicle (100 μL; control group) or Lis (100 μL of vehicle containing 200 μg Lis; Lis group). In another set of animals, mice received a single Entresto dose by gavage 30 min prior to injection of the radiotracer, together with the vehicle (100 μL; Entresto group) or Lis (100 μL of vehicle containing 200 μg Lis; Entresto + Lis group). For animal treatment, Entresto (24/26 sacubitril/valsartan) pills were ground to a fine powder in a mortar, divided, and suspended in tab water to individual 12 mg/200 mL doses per animal. Animals were euthanized 5 min pi, and blood was collected and immediately placed in pre-chilled polypropylene vials containing EDTA on ice. Samples were centrifuged at 2000× *g* at 4 °C for 10 min, then the plasma was collected and mixed with an equal volume of MeCN and centrifuged again for 10 min at 15,000× *g* at 4 °C. The supernatant was collected and concentrated to a small volume under a gentle N_2_ flux at 40 °C, diluted with physiological saline (400 μL) and filtered through a Millex GV filter (0.22 μm). Suitable aliquots of the filtrate were analyzed by RP-HPLC on a Symmetry Shield RP18 (5 μm, 3.9 mm × 20 mm) column (Waters, Germany), eluted at a flow rate of 1 mL/min and adopting gradient system 2: 100% A/0% B to 70% A/30% B in 30 min; A = 0.1% TFA in H_2_O, and B = MeCN (system 2). The elution time (*t*_R_) of the intact radioligand was determined by co-injection with a sample of the labelling reaction solution.

#### 4.3.2. Tumor Induction in SCID Mice

A suspension of freshly harvested AsPC-1 (~150 μL, 5 × 10^6^) or MiaCapa-2 (~150 μL, 3.5 × 10^6^) cells in normal saline was subcutaneously inoculated in the flanks of 6-week-old SCID mice (NCSR “Demokritos” Animal House; group A = 11 mice, 20.34 ± 1.63 g body weight; group B = 4 mice, 22.81 ± 0.25 g body weight). After 3–4 weeks, palpable tumors (group A, AsPC-1: 0.23 ± 0.11 g; group B: MiaCapa-2: 0.14 ± 0.05 g) had developed at the inoculation sites, and biodistribution was performed.

#### 4.3.3. Biodistribution in SCID Mice Bearing AsPC-1 and MiaCapa-2 Xenografts

At the day of biodistribution, a bolus of [^99m^Tc]Tc–DT1 (185 kBq, 10 pmol total peptide, in vehicle: saline/EtOH = 9:1 *v*/*v*) was intravenously injected in the tail of group A mice, together with the vehicle (100 μL; control group: four mice). The rest of group A mice were treated with Entresto 30 min prior to radioligand co-injection with Lis (200 μg Lis dissolved in 100 μL vehicle; Entresto + Lis-group: four mice), or with Lis plus excess NT (200 μg Lis and 100 μg NT dissolved in 100 μL vehicle; block group: three mice). Animals were euthanized at 4 h pi, and blood samples, organs of interest, and AsPC-1 tumors were dissected, weighed, and counted in the gamma counter. Biodistribution data was calculated as percent of injected activity per gram tissue (% IA/g) with the aid of suitable standards of the injected activity, using the Microsoft Excel program. Results represent mean values ± SD, *n* = 4.

Mice of group B (four animals with MiaCapa-2 tumors) were treated with the Entresto + Lis combination, as described above, and the same protocol was followed.

#### 4.3.4. Statistical Analysis

For statistical analysis of biological results, a two-way ANOVA with multiple comparisons was used, applying Tukey’s post-hoc analysis (GraphPad Prism Software, San Diego, CA, USA); *p* values of <0.05 were considered to be statistically significant.

All animal studies were performed in compliance to European guidelines in supervised and licensed facilities (EL 25 BIO 021), whereas the study protocols were approved by the Department of Agriculture and Veterinary Service of the Prefecture of Athens (revised protocol number 1609 approved on 24 April 2019 for the stability studies and revised protocol number 1610 approved on 24 April 2019 for biodistribution and imaging studies).

## 5. Conclusions

Pancreatic cancer, and in particular PDAC, is a devastating disease with five-year survival below 5%. Typically, cancer has already spread without symptoms until the time of diagnosis, and prognosis is poor. Consequently, molecular tools for diagnosis and therapy—theranostics—of PDAC are urgently needed to improve survival rates for patients. In the present work, we have evaluated the NT(7–13)-based radiotracer [^99m^Tc]Tc–DT1 in NTS1R-positive pancreatic cell and mice models, given that overexpression of the NTS1R target has been documented in PDAC. We selected AsPC-1 cells amongst a panel of human pancreatic cancer cell lines as our study model, based on the superior NTS1R-specific uptake of [^99m^Tc]Tc–DT1 in these cells. Next, we were able to markedly stabilize [^99m^Tc]Tc–DT1 in mice circulation through the administration of two clinically established inhibitors, namely Entresto (for NEP inhibition) and Lis (for ACE inhibition). Last but not least, we could induce notable increases in the NTS1R-specific uptake of [^99m^Tc]Tc–DT1 in AsPC-1 tumors xenografted in mice compared with controls. These findings are in favor of clinical translation of the protease-inhibition concept in PDAC patients.

## Figures and Tables

**Table 1:** Stabilities of [^99m^Tc]Tc–DT1 and [^99m^Tc]Tc–DT5 in peripheral mouse blood 5 min post-injection (pi) in untreated controls and in animals treated with Entresto, lisinopril (Lis), or their combination.

	Control	Entresto	Lis	Entresto+Lis
[^99m^Tc]Tc–DT1	1.8 ± 0.8 (*n* = 4)	5.5 ± 3.9 (*n* = 5)	18.8 ± 2.5 (*n* = 3)	63.8 ± 7.5 (*n* = 3)
[^99m^Tc]Tc–DT5	1.2 ± 0.2 (*n* = 3)	2.0 ± 0.6 (*n* = 3)	28.7 ± 3.6 (*n* = 3)	70.2 ± 4.9 (*n* = 3)

Data represents the mean percentage of intact radioligand ± SD; *n* of experiments are shown in parentheses.

**Table 2:** Biodistribution data for [^99m^Tc]Tc–DT1, expressed as % IA/g mean ± SD in AsPC-1 xenograft-bearing SCID mice at 4 h pi, without or after treatment with Entresto + Lis.

	[^99m^Tc]Tc–DT1: 4 h pi				
	Controls ^1^		Entresto + Lis ^2^		Block ^3^
Blood	0.07 ± 0.01		0.08 ± 0.02		0.09 ± 0.01
Liver	0.44 ± 0.05		0.67 ± 0.06		0.55 ± 0.06
Heart	0.08 ± 0.01		0.11 ± 0.02		0.18 ± 0.11
Kidneys	4.18 ± 3.80	⇤ *p* < 0.0001 ⇥	6.81 ± 1.74	⇤ *p* < 0.001 ⇥	4.15 ± 1.75
Stomach	0.48 ± 0.26		0.57 ± 0.14		2.75 ± 1.63
Intestines	0.65 ± 0.04		2.13 ± 0.24		1.07 ± 0.01
Spleen	0.22 ± 0.05		0.58 ± 0.35		0.31 ± 0.07
Muscle	0.03 ± 0.01		0.06 ± 0.03		0.07 ± 0.06
Lungs	0.18 ± 0.03		0.62 ± 0.35		0.41 ± 0.07
Pancreas	0.05 ± 0.01		0.10 ± 0.01		0.08 ± 0.00
Tumor	1.25 ± 0.14	⇤ *p* < 0.0001 ⇥	7.05 ± 0.80	⇤ *p* < 0.0001 ⇥	0.74 ± 0.01

All animals were injected with 185 kBq/10 pmol peptide; ^1^ Control mice group (*n* = 4) with untreated animals; ^2^ Entresto + Lis mice group (*n* = 4) with animals receiving 12 mg Entresto per os 30 min prior to radiotracer co-injection, together with 200 µg Lis to in situ to inhibit NEP and ACE, respectively. ^3^ Block mice group (*n* = 3), with animals co-injected with 100 µg NT for in vivo NTS1R blockade, in addition to being treated with the Entresto + Lis combination.

## Supplementary Materials

Supplementary materials can be found at https://www.mdpi.com/1422-0067/21/21/7926/s1. Figure S1: Structures of the ACE inhibitor Lis and of sacubitril (AHU377) found in the drug Entresto, releasing the active substance sacubitrilat (LBQ657) in vivo upon ester-hydrolysis by esterases; sacubitrilat is a potent and specific NEP inhibitor. Table S1: Cellular uptake, with membrane-bound and internalized activity fragments shown separately, of [^99m^Tc]Tc–DT1 at 1 h incubation across cell lines, expressed as mean ± SD, including specific and non-specific portions. Table S2: Biodistribution data for [^99m^Tc]Tc–DT1, expressed as % IA/g mean ± SD, in MiaPaca-2 xenograft-bearing SCID mice treated with Entresto + Lis at 4 h pi.
