# Supplementary material for: Optimizing the Profile of [99mTc]Tc–NT(7–13) Tracers in Pancreatic Cancer Models by Means of Protease Inhibitors
Source: Int J Mol Sci. 2020 Oct 26;21(21):7926. doi: 10.3390/ijms21217926 (PMC7663772; doi:10.3390/ijms21217926)
Supplement: Supplementary file 1 [file ijms-21-07926-s001.pdf]

International Journal of *Molecular Sciences*

# Supplemental Materials

# Optimizing the Profile of [$^{99m}$Tc]Tc–NT(7–13) Tracers in Pancreatic Cancer Models by Means of Protease Inhibitors

**Panagiotis Kanellopoulos [1,2,*], Berthold A. Nock [1], Eric P. Krenning [3] and Theodosia Maina [1,*]**

1 Molecular Radiopharmacy, INRASTES, NCSR "Demokritos", 15341 Athens, Greece; nock_berthold.a@hotmail.com
2 Molecular Pharmacology, School of Medicine, University of Crete, Heraklion, 70013 Crete, Greece
3 Cyclotron Rotterdam BV, Erasmus MC, 3015 CE Rotterdam, The Netherlands; erickrenning@gmail.com
* Correspondence: kanelospan@gmail.com (P.K.); maina_thea@hotmail.com (T.M.); Tel.: +30-210-650-3891 (P.K.); +30-210-650-3908 (T.M.)

(**a**)

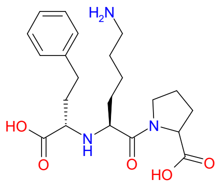


(**b**)

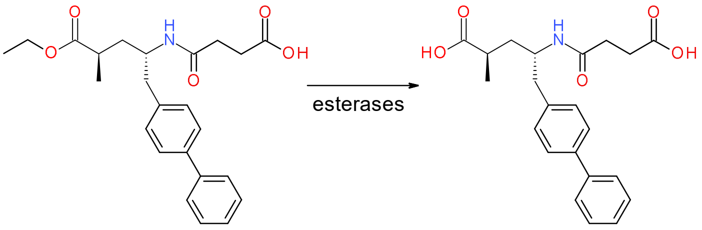


**Figure S1.** Structure of (**a**) the angiotensin converting enzyme (ACE)-inhibitor lisinopril (Lis) and (**b**) the structure of sacubitril (AHU377) found in the drug Entresto, releasing the active substance sacubitrilat (LBQ657) in vivo upon ester-hydrolysis by esterases; sacubitrilat is a potent and specific inhibitor of neprilysin (NEP).

**Table S1.** Cellular uptake (membrane bound and internalized of total-added activity) of [$^{99m}$Tc]Tc-DT1 at 1 h incubation across cell lines, expressed as mean±SD, including specific and non-specific portions.

| Cell line | | MB[1] | I[2] |
|---|---|---|---|
| AsPC-1 | specific | 0.98±0.42 | 14.11±2.28 |
| | non-specific[3] | 0.67±0.23 | 0.88±0.59 |
| PANC-1 | specific | 0.76±0.39 | 7.30±2.41 |
| | non-specific | 0.44±0.20 | 0.29±0.12 |
| MiaPaca-2 | specific | 0.37±0.23 | 2.46±0.23 |
| | non-specific | 0.87±0.49 | 0.50±0.36 |
| Capan-1 | specific | 0.10±0.01 | 0.28±0.13 |
| | non-specific | 0.20±0.02 | 0.11±0.02 |

[1] MB, membrane bound fraction; [2] I, internalized fraction; non-specific values determined by incubation in the presence of 1 μM NT; these values were subtracted from the respective measured MB/I fractions to calculate the respective specific values.

**Table S2.** Biodistribution data for [$^{99m}$Tc]Tc-DT1, expressed as %IA/g mean±SD, in MiaPaca-2 xenograft-bearing SCID mice treated with the Entresto+Lis combination at 4 h pi.

| Tissue | [$^{99m}$Tc]Tc-DT1 – 4 h pi |
|---|---|
| | Entresto+Lis[2] |
| Blood | 0.06±0.01 |
| Liver | 0.57±0.04 |
| Heart | 0.08±0.02 |
| Kidneys | 5.86±0.14 |
| Stomach | 0.37±0.05 |
| Intestines | 2.43±0.6 |
| Spleen | 0.49±0.03 |
| Muscle | 0.03±0.01 |
| Lungs | 0.43±0.04 |
| Pancreas | 0.1±0.02 |
| Tumor | 1.97±0.25 |

All animals were injected with 185 kBq/10 pmol peptide; Entresto+Lis mice group (n = 4) with animals receiving 12 mg Entresto per os 30 min prior to radiotracer co-injection together with 200 μg Lis to in situ inhibit NEP and ACE, respectively.
